# Supplementary material for: Achievement of Low-Density Lipoprotein Cholesterol Targets in Cardiac Rehabilitation: Impact of the 2019 ESC/EAS Dyslipidaemia Guidelines
Source: J Clin Med. 2022 Nov 29;11(23):7057. doi: 10.3390/jcm11237057 (PMC9740039; doi:10.3390/jcm11237057)
Supplement: Supplementary file 1 [file jcm-11-07057-s001.zip › jcm-2021032-supplementary.pdf]

Journal of
Clinical Medicine

MDPI

Supplementary Material

Suppl. Table S1. Therapy at CR entry stratified after discharge time period.

	Group A [593]	Group B [282]	p
Statins	569 (96)	273 (97)	0.67
High potent Statins	545 (96)	270 (99)	**0.020**
Statins and Ezetimibe	36 (6)	50 (18)	**<0.001***
Statin Dose			**<0.001***
12.5 %MDD	9 (2)	1 (0)	0.18
25 %MDD	53 (9)	27 (10)	0.89
50 %MDD	440 (77)	198 (73)	0.14
75 %MDD	1 (0)	2 (1)	0.25
100 %MDD	62 (11)	44 (16)	**0.044***
…≥50% MDD	503 (85)	244 (87)	0,81
Statin associated symptoms (%)	0 (0)	5 (2)	**0.0035***
Ezetimibe (%)	38 (6)	52 (18)	**<0.001***
PCSK9-Inhib. (%)	2 (0)	4 (1)	0.089

Categorical and interval scaled variables are described as n with %; Indication for significance= p Overall: 0.05 (indicated by bold letters and *); MDD: Maximum of daily dose. MDD: maximal daily dose. 40mg Rosuvastatin and 80 mg Atorvastatin is defined as 100% MDD.

Supple Table S2. Therapy at CR discharge stratified after discharge time period.

	Group A [593]	Group B [282]	p
Statins	575 (97)	277 (98)	0.37
High potent Statins	555 (96)	272 (98)	0.34
Statins and Ezetimibe	103 (17)	145 (51)	**<0.001***
Statin Dose			**<0.001***
12.5 %MDD	15 (3)	4 (1)	0.33
25 %MDD	83 (14)	28 (10)	0.094
50 %MDD	422 (74)	188 (68)	0.088
75 %MDD	1 (0)	2 (1)	0.25
100 %MDD	51 (9)	55 (20)	**<0.001***
…≥50% MDD	474 (80)	245 (87)	**0.046**
Statin associated symptoms (%)	9 (2)	17 (6)	**<0.001***
Ezetimibe (%)	105 (18)	145 (51)	**<0.001***
PCSK9-Inhib. (%)	3 (1)	5 (2)	0.12

Categorical and interval scaled variables are described as n with %; Indication for significance= p Overall: 0.05 (indicated by bold letters and *); MDD: Maximum of daily dose. 40mg Rosuvastatin and 80 mg Atorvastatin is defined as 100% MDD.

Supple Table S3. Logistic Regression Weights for Statin dosage at CR discharge.

	Coefficient/Estimate	95% Confidence Interval	p
**Intercept**	-7.5	[-10.9, -4.1]	**<0.001***
Age	0.0091	[-0.018, 0.036]	0.51
Male Sex	-0.067	[-0.79, 0.66]	0.86
Hypertension	0.57	[-0.12, 1.2]	0.10
Dyslipidaemia	1.8	[0.89, 2.8]	**<0.001***
Smoking History	0.066	[-0.56, 0.69]	0.84

Therapy PCI	2.0	[-0.024, 4.0]	0.053
Therapy CABG	2.0	[-0.062, 4.2]	0.057
Group B	0.65	[0.074, 1.2]	**0.027***
Rehabilitation Duration	0.0027	[-0.012, 0.017]	0.72
Family History for Cardiovascular Events	0.059	[-0.52, 0.64]	0.84

Multivariate quantile regression model identifying factors associated with statin dosage at CR discharge. Positive coefficients/estimates indicate a positive correlation between above mentioned independent factors and statin dosage at CR discharge. Indication for significance= p Overall: 0.05 (indicated by bold letters and *). CABG: Coronary artery bypass surgery; PCI: Percutaneous coronary intervention.

Supple Table S4. Logistic Regression Weights for new Ezetimibe prescription at CR discharge.

	**Coefficient/Estimate**	**95% Confidence Interval**	**p**
**Intercept**	-1.83	[-3.5, -0.18]	**0.030**
Age	-0.0080	[-0.025, 0.0092]	0.36
Male Sex	-0.40	[-0.85, 0.044]	0.077
Hypertension	0.13	[-0.27, 0.54]	0.51
Dyslipidaemia	0.66	[0.24, 1.1]	**0.0018***
Smoking History	0.50	[0.082, 0.91]	**0.019***
Therapy PCI	-0.16	[-0.78, 0.46]	0.61
Therapy CABG	-0.041	[-0.80, 0.71]	0.91
Group B	1.3	[0.90, 1.6]	**<0.001***
Rehabilitation Duration	-0.0010	[-0.0084, 0.0064]	0.79
Family History for Cardiovascular Events	0.13	[-0.25, 0.50]	0.51

Multivariate quantile regression model identifying factors associated with ezetimibe prescription at CR discharge. Positive coefficients/estimates indicate a positive correlation between above mentioned independent factors and ezetimibe prescription at CR discharge. Indication for significance= p Overall: 0.05 (indicated by bold letters and *). CABG: Coronary artery bypass surgery; PCI: Percutaneous coronary intervention.

Flowchart of patient enrolment

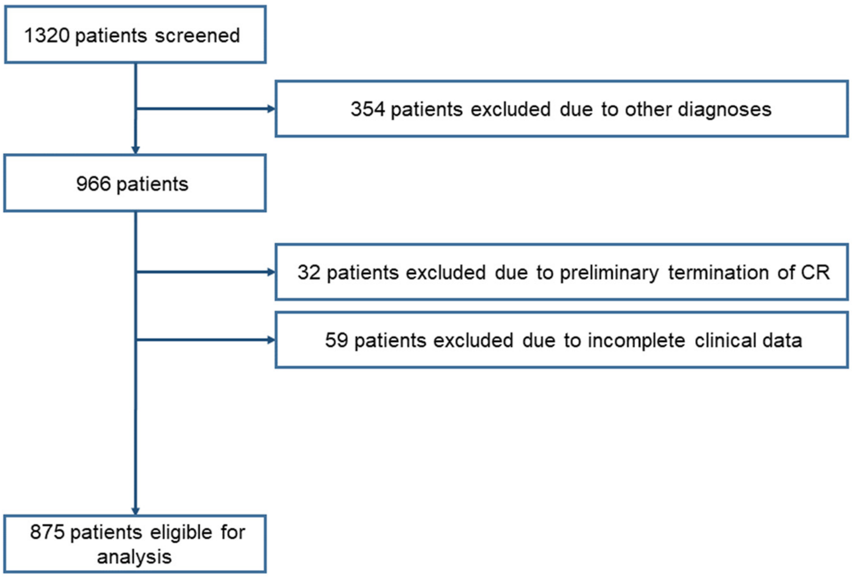

Supple Figure S1. Flowchart of patient enrolment.

LDL-C distribution at CR entry and discharge referencing the ESC/EAS 2016 and 2019 guideline targets

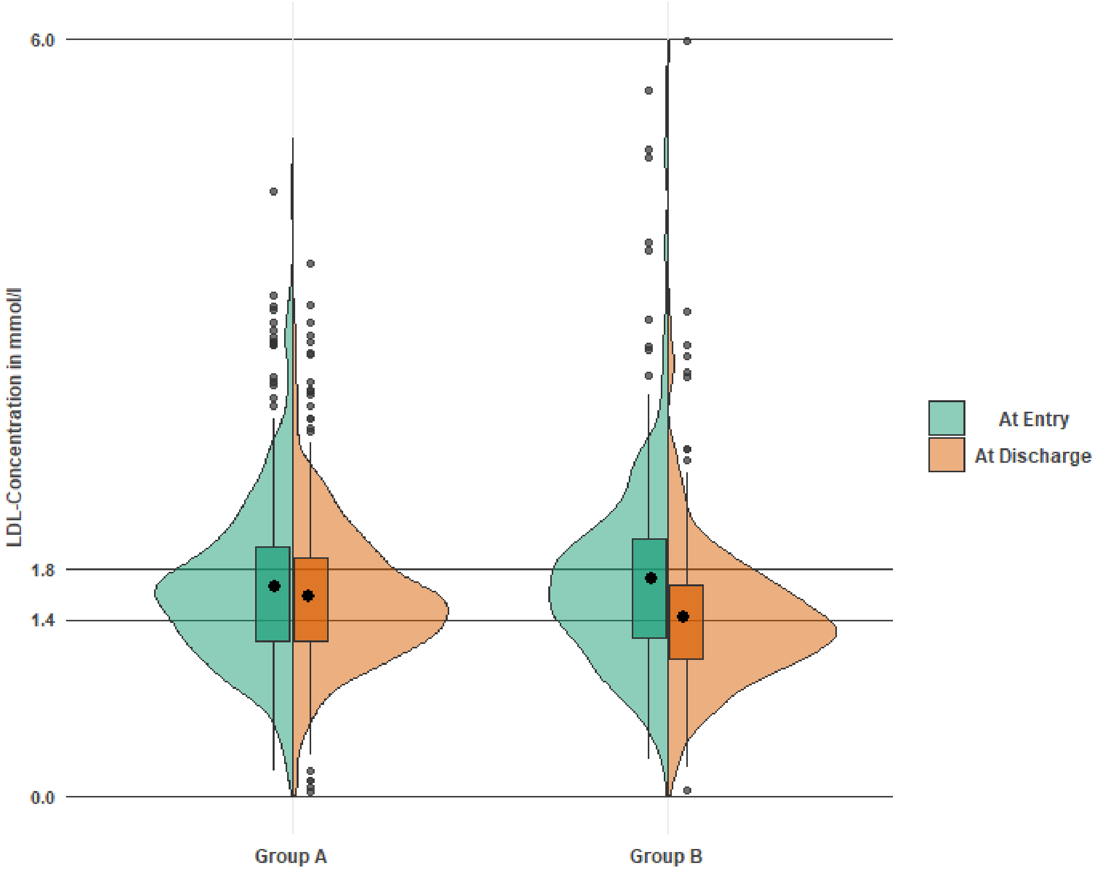

Supple Figure S2. LDL-C distribution at CR baseline and discharge in the entire cohort, group A and group B shown in split violin plot referencing the ESC/EAS 2016 and 2019 guideline targets. The boxes represent the first to third quartile, the black point shows the median. The vertical lines represent the 95th percentile, the small points show outliers.
